# Sodium–Glucose Cotransporter 2 and Glucose Levels Affect Clear Cell Renal Cell Carcinoma Progression

**DOI:** 10.3390/ijms26125501

**Published:** 2025-06-08

**Authors:** Yujiro Nagata, Ikko Tomisaki, Hisami Aono, Nguyen Thu Quynh, Eiji Kashiwagi, Naohiro Fujimoto

**Affiliations:** 1 Department of Urology, School of Medicine, University of Occupational and Environmental Health, Kitakyushu 807-8555, Japan; ikko@med.uoeh-u.ac.jp (I.T.); hisami-aono@med.uoeh-u.ac.jp (H.A.); quynh@med.uoeh-u.ac.jp (N.T.Q.); ekashiwagi@med.uoeh-u.ac.jp (E.K.); n-fuji@kurate-hp.com (N.F.); 2 Department of Urology, Kurate Hospital, Kurate 807-1311, Japan

**Keywords:** clear cell renal cell carcinoma, SGLT2, glucose, tumor progression, *von Hippel–Lindau* gene

## Abstract

The biological significance of sodium–glucose cotransporter 2 (SGLT2) in clear cell renal cell carcinoma (ccRCC) has yet to be elucidated. In this study, we aimed to determine the role of SGLT2 in ccRCC tumor progression. The human ccRCC line KMRC-1, which contains a von Hippel–Lindau (*VHL*) gene mutation, was used to assess the effects of the SGLT2 inhibitor (SGLT2i) dapagliflozin on proliferation and migration in media containing different glucose concentrations (25, 12.5, or 5 mM). Dapagliflozin significantly reduced cell proliferation and migration in 25 mM glucose medium. Similarly, SGLT2 knockdown involving short hairpin RNA lentiviral transfection significantly decreased cell viability, migration, and colony formation compared with the control subline in 25 mM glucose medium. Moreover, tumor progression was inhibited in the media with low glucose concentrations. Remarkably, 2 µM dapagliflozin inhibited the progression of ccRCC at concentrations as low as 5 mM (normoglycemic model) glucose medium as well as 25 mM (severe glycemia model) glucose medium. In addition, dapagliflozin treatment significantly enhanced the apoptosis of ccRCC cells. Our findings demonstrate that SGLT2 impacts the progression of ccRCC with the *VHL* mutation. In light of the above findings, SGLT2is, which exert the dual effects of SGLT2 blockade and glycemic control, may represent a novel therapeutic agent, particularly in patients with ccRCC who suffer from concurrent diabetes mellitus. To the best of our knowledge, this is the first preclinical study demonstrating the impact of SGLT2 inhibition on the progression of ccRCC with the *VHL* mutation.

## 1. Introduction

Renal cell carcinoma (RCC) is a common form of cancer, with an estimated global incidence of approximately 435,000 cases and 156,000 deaths in 2022 [1]. The most frequent histological subtype of RCC is clear cell RCC (ccRCC), and approximately 25–30% of patients with ccRCC have distant metastases at the time of diagnosis [2]. Recent advancements in targeted therapies and immune checkpoint inhibitors, including programmed death-1, programmed death-ligand 1, and cytotoxic T lymphocyte antigen-4 inhibitors, have significantly improved the survival odds of patients with advanced or metastatic ccRCC [2]. However, because of the inherent limitations of these treatments, such as low response rates, the acquisition of drug resistance, and severe adverse events, ccRCC remains deadly. There is, therefore, an urgent need to develop novel therapeutic agents to target this type of cancer.

ccRCC is characterized by the functional loss of the von Hippel–Lindau (VHL) complex. A large-scale genomic analysis of sporadic ccRCC performed by the Cancer Genome Atlas Research Network revealed functional loss of the VHL complex in 90% of ccRCC cases due to mutations in *VHL* (a tumor suppressor gene), DNA methylation of the *VHL* promoter, or mutations in *TCEB1* encoding elongin C (a component of the VHL complex) [3,4]. From these findings, it is evident that *VHL* alterations act as driver mutations directly contributing to ccRCC development. Loss of function of the VHL complex inhibits hypoxia-inducible factor (HIF)-2α degradation, leading to metabolic reprogramming mimicking the response to a hypoxic environment [5]. This metabolic adaptation requires high levels of glucose uptake to sustain energy production via anaerobic respiration.

Diabetes mellitus (DM) is a systemic metabolic disease characterized by hyperglycemia. The disease can affect any organ, resulting in a variety of complications, such as nephropathy [6]. Epidemiologically, DM is associated with increased incidence and mortality of several malignancies [7,8,9], including RCC [10,11,12]. Cancer cells require substantial amounts of glucose to support their high rate of glycolysis, which is necessary to fuel cell growth and division [13]. There is limited research on the relationship between DM, glycemic control, and ccRCC progression. Determining the effect of glucose concentration on the progression of ccRCC may, therefore, contribute to the development of novel therapeutic strategies, particularly for patients with ccRCC who suffer from concurrent DM.

Glucose enters cells via glucose transporters. *SLC5A2* encodes the sodium–glucose cotransporter 2 (SGLT2) protein, which is almost exclusively expressed in proximal tubular epithelial cells of the kidneys and is responsible for the reabsorption of nearly 90% of filtered glucose. SGLT2 inhibitors (SGLT2is), initially developed as therapy for type 2 DM, increase urinary glucose excretion and, thereby, reduce blood glucose concentrations. SGLT2is, particularly dapagliflozin and empagliflozin, have recently been reported to be associated with a decreased incidence of RCC in patients with type 2 DM [14]. Furthermore, SGLT2is have demonstrated potential anticancer properties in various cancer types [15]. Notably, the results of an in vitro study showed that SGLT2is suppressed RCC progression; however, the study was restricted to RCC cell lines with wild-type *VHL* [16]. Moreover, immunohistochemical studies on surgically excised ccRCC tissue showed SGLT2 positivity in 68% of cases, and increased SGLT2 expression was associated with shorter overall survival [17].

Collectively, these findings suggest a relationship between SGLT2, glycemic control, and ccRCC progression. The aim of the present study was, therefore, to evaluate the impact of SGLT2 and glucose concentration levels on the progression of ccRCC with the *VHL* mutation using in vitro models that were not subject to the many potential confounders linked to DM in vivo.

## 2. Results

### 2.1. The Presence of SGLT2 in Human ccRCC Cells

**Figure 1:**
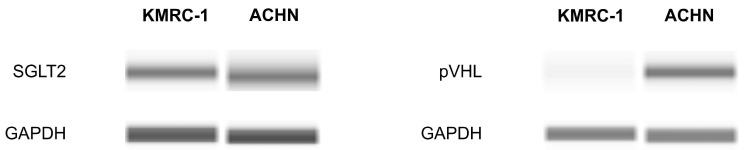
Determination of sodium–glucose cotransporter 2 (SGLT2) and von Hippel–Lindau protein (pVHL) presence in KMRC-1 and ACHN cells using Western blotting. GAPDH served as an internal control. Images are representative of three independent experiments.

To begin our experiment, the presence of the SGLT2 protein in ccRCC cells was investigated using Western blotting. SGLT2 was detected in both a *VHL* mutation ccRCC line (KMRC-1) and a *VHL* wild-type RCC cell line (ACHN). As anticipated, KMRC-1 cells displayed SGLT2 but not von Hippel–Lindau protein (pVHL) (Figure 1).

### 2.2. The Impact of SGLT2 Inactivation and Glucose Concentrations on ccRCC Cell Progression

**Figure 2:**
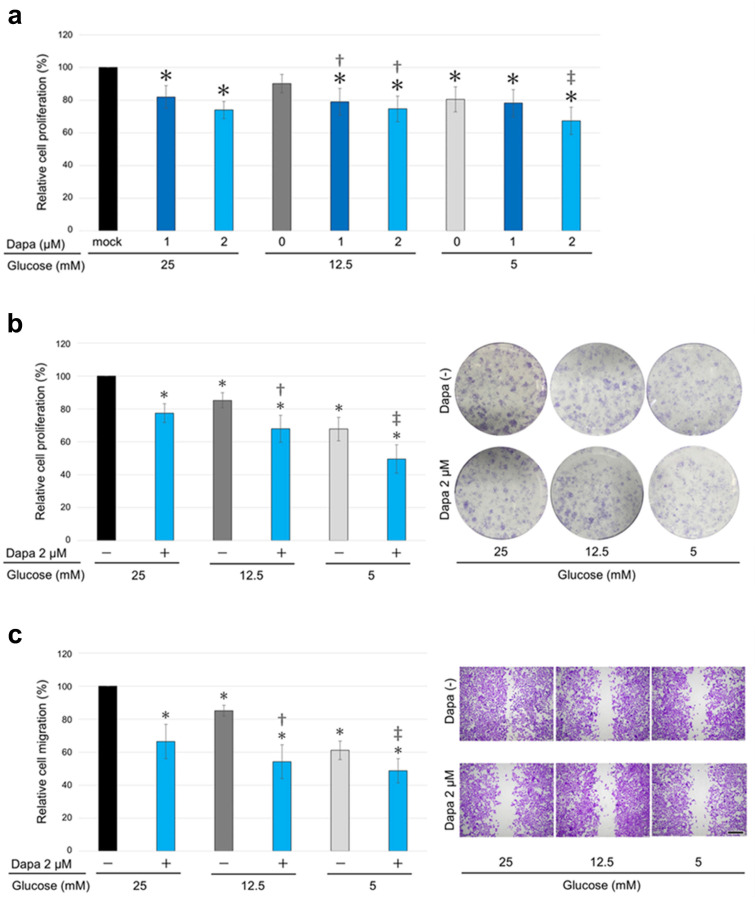
The effects of dapagliflozin and different glucose concentrations on clear cell renal cell carcinoma (ccRCC) progression. (**a**) MTT assay of KMRC-1 cells cultured in media containing 25, 12.5, or 5 mM glucose supplemented with 10% fetal bovine serum (FBS) and treated with dimethyl sulfoxide (DMSO; mock) or dapagliflozin (dapa; 1 or 2 µM) for 96 h. (**b**) Clonogenic assay in KMRC-1 cells cultured in medium containing 25, 12.5, or 5 mM glucose supplemented with 10% FBS and treated with DMSO (mock) or 2 µM dapagliflozin for 10 days. (**c**) Scratch wound healing assay in KMRC-1 cells cultured in medium containing 25, 12.5, or 5 mM glucose without FBS supplementation and treated with DMSO (mock) or 2 µM dapagliflozin for 24 h after scratching. Cell viability, colony number (≥20 cells), and the width of the wound area are presented relative to those of mock treatment with 25 mM glucose and represent the mean (±SD) of three independent experiments. Scale bar: 500 μm. * *p* < 0.05 (vs. mock treatment with 25 mM glucose), ^†^ *p* < 0.05 (vs. mock treatment with 12.5 mM glucose), and ^‡^ *p* < 0.05 (vs. mock treatment with 5 mM glucose).

The viability (via MTT assay), colony formation (via clonogenic assay), and migration (via a scratch wound healing assay) of KMRC-1 cells cultured with the SGLT2i dapagliflozin were compared. The results of the MTT assay demonstrated that dapagliflozin treatment significantly reduced cell viability in a dose-dependent manner (Figure 2a). Similarly, the results of the clonogenic assay showed that dapagliflozin significantly inhibited colony formation (Figure 2b), and the wound healing assay revealed that dapagliflozin significantly inhibited cell migration (Figure 2c).

Next, the impact of different glucose concentrations on ccRCC cell progression was assessed. KMRC-1 cells were cultured in media containing 25 mM (450 mg/dL: severe glycemia model), 12.5 mM (225 mg/dL: mild glycemia model), or 5 mM (90 mg/dL: normoglycemic model) glucose supplemented with 10% fetal bovine serum (FBS). The medium containing 5 mM glucose inhibited ccRCC cell progression (Figure 2a–c). Furthermore, dapagliflozin inhibited cell viability, colony formation, and migration across all tested glucose concentrations, including low glucose concentrations (Figure 2a–c).

**Figure 3:**
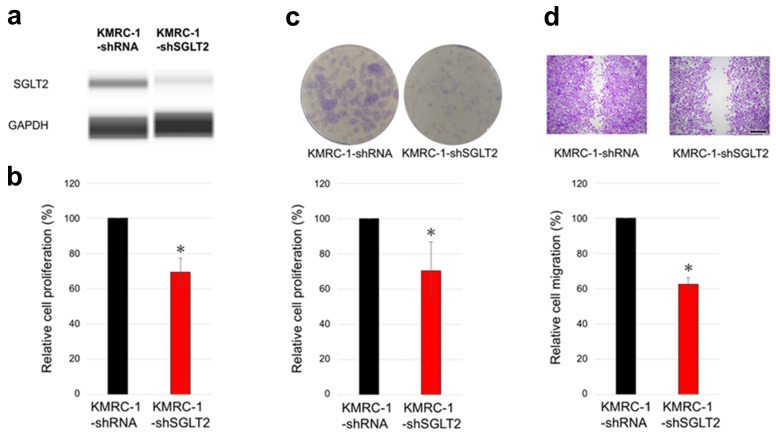
The effects of SGLT2 knockdown on ccRCC cells. (**a**) Western blotting for SGLT2 in KMRC-1-control-short hairpin RNA (shRNA) and KMRC-1-SGLT2-shRNA (knockdown) sublines. GAPDH, loading control. Images are representative of three independent experiments. (**b**) MTT assay in KMRC-1-control-shRNA and KMRC-1-SGLT2-shRNA sublines cultured in medium supplemented with 10% FBS for 96 h. (**c**) Clonogenic assay of KMRC-1-control-shRNA and KMRC-1-SGLT2-shRNA sublines cultured for 10 days in medium supplemented with 10% FBS. (**d**) Scratch wound healing assay in KMRC-1-control-shRNA and KMRC-1-SGLT2-shRNA cells cultured in serum-free medium for 24 h after scratching. Cell viability, number of colonies (≥20 cells), and wound width are presented relative to those of the control-shRNA subline and represent the mean (±SD) of three independent experiments. Scale bar: 500 μm. * *p* < 0.05 (vs. the control-shRNA subline).

The ccRCC cell progression in KMRC-1 sublines stably expressing KMRC-1-control-short hairpin RNA (shRNA) with KMRC-1-SGLT2-shRNA (knockdown) was compared. First, the results of Western blot analysis confirmed the successful knockdown of SGLT2, as evidenced by a considerable reduction in the presence of the SGLT2 protein in the knockdown subline (Figure 3a). Thereafter, as anticipated, SGLT2 knockdown resulted in significantly decreased cell viability (Figure 3b), colony formation (Figure 3c), and cell migration (Figure 3d) compared with the control.

### 2.3. The Impact of SGLT2 Inhibition on the Apoptosis of ccRCC Cells

**Figure 4:**
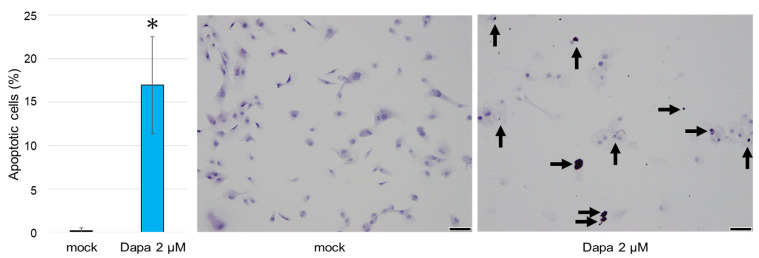
The effects of dapagliflozin on the apoptosis of ccRCC cells monitored using a TUNEL assay. The arrows indicate TUNEL-positive staining. TUNEL-positive cells were rarely observed in ccRCC cells treated with the mock. Each value represents the mean (±SD) from three independent experiments. Scale bar: 50 μm. * *p* < 0.05 (vs. mock treatment).

A TUNEL assay was performed on ccRCC cells to detect apoptotic cells. Following treatment with 2 µM dapagliflozin for 96 h, a greater number of TUNEL-positive cells were observed in cells treated with dapagliflozin compared to those treated with the mock (Figure 4).

### 2.4. SLC5A2 Expression in ccRCC Tissues

**Figure 5:**
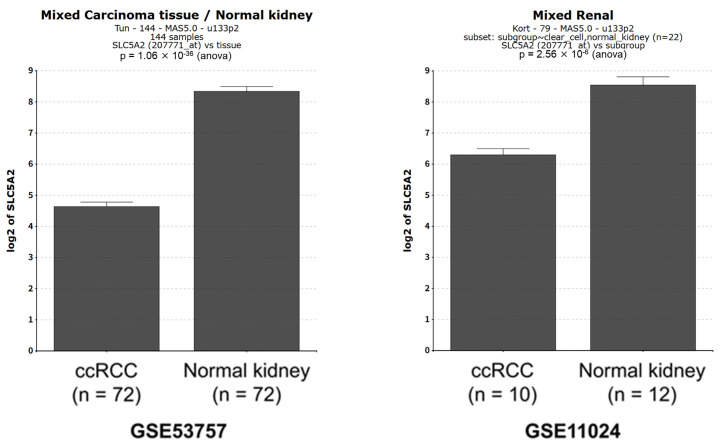
Expression levels of *SLC5A2* in ccRCC tissue versus normal kidney tissue. The Gene Expression Omnibus repository datasets (GSE53757 and GSE11024) were obtained from the R2 Genomics Analysis and Visualization Platform. Differences were analyzed using Student’s *t*-test.

A publicly available database was utilized to analyze *SLC5A2* expression in ccRCC. Data obtained from GSE53757 [18] and GSE11024 [19] highlighted the expression of *SLC5A2* in ccRCC tissues. However, the expression levels were significantly lower compared with those in corresponding normal kidney tissues (Figure 5).

## 3. Discussion

It has been recently reported that SGLT2, which is predominantly expressed in kidney tissue, is also expressed in various cancers [20], including ccRCC [17]. The results of in vitro studies suggest that SGLT2is inhibit the progression of several malignancies, including colon cancer [21], prostate and lung cancer [22], liver cancer [23], breast cancer [24,25], pancreatic cancer [26], thyroid cancer [27], osteosarcoma [28], and renal cancer [16]. The anticancer mechanisms of SGLT2is vary, including the inhibition of glucose uptake in cancer cells, the Wnt/β-catenin pathway, cell adhesion, angiogenesis, and DNA and RNA synthesis and activation of the AMPK pathway [29]. Remarkably, the results of preclinical studies demonstrate that dapagliflozin inhibits RCC growth in vitro and in vivo [16,30], and the results of recent epidemiological studies reveal the significant impact of SGLT2i treatment on the progression of various malignancies [20]. Furthermore, the results of propensity score-matched population-based studies have demonstrated an association between SGLT2i use and reduced RCC risk, primarily in patients with type 2 DM [14,31]. Notably, Lin et al. demonstrated a type disparity in SGLT2is, in which dapagliflozin was associated with a significantly lower risk of incident RCC and had the lowest hazard ratio of all SGLT2i types under investigation [31]. Collectively, these findings suggest that SGLT2is, particularly dapagliflozin, are potential therapeutics for RCC. However, the authors of the preclinical studies conducted to date have only evaluated RCC cell lines with wild-type *VHL* [16,30], with some of the cells exhibiting non-ccRCC histology [32]. Furthermore, data obtained from large population-based databases are limited to patients with DM, with insufficient adjustment for DM severity and duration, and lack information on the histopathological subtype of RCC. We addressed these limitations in our study by evaluating the impact of SGLT2 on the progression of a *VHL* mutant ccRCC cell line in which the mutation had resulted in metabolic reprogramming of glucose metabolism. The use of this in vitro model meant that the cells were not subjected to the wide range of potential confounders linked to DM in vivo.

Our evaluation of the effects of SGLT2 inhibition on the viability, colony formation, and migration of *VHL* mutant ccRCC cells revealed that the SGLT2i dapagliflozin inhibited progression of ccRCC partly due to the induction of apoptosis. These inhibitory effects were further confirmed by the inhibition of ccRCC cell progression following SGLT2 knockdown. Our findings, therefore, suggest that the inhibition of ccRCC progression is induced by SGLT2is. To the best of our knowledge, this is the first preclinical study demonstrating the impact of SGLT2 inhibition on the progression of ccRCC in cells with the *VHL* mutation. Furthermore, the expression of *SLC5A2*, which encodes SGLT2, has been observed in ccRCC tissues. Based on the above findings, SGLT2 may represent a potential therapeutic target in ccRCC. In addition, future studies involving the use of other ccRCC cell lines with *VHL* mutations are warranted not only to validate our results but to also elucidate the molecular mechanisms underlying SGLT2-mediated ccRCC progression.

One of the hallmarks of cancer cells is that they require more glucose than benign cells. This distinction is the result of the reprogramming of glucose metabolism to favor glycolysis, even in the presence of adequate oxygen, known as the Warburg effect [13,33]. To compensate for the up to 18-fold lower efficiency of adenosine triphosphate production resulting from glycolysis relative to mitochondrial oxidative phosphorylation, cancer cells require high levels of glucose uptake and utilization. Specifically, ccRCC cells contain abundant cytoplasmic glycogen and lipids [34] as a consequence of the metabolic reprogramming partially induced by VHL complex deficiency and HIF-2α stabilization, and HIF-2α induces glucose transporter-1 overexpression in ccRCC [35]. Interestingly, the authors of an in vitro study reported that *VHL* mutant ccRCC cells showed a tendency to contain large amounts of carbohydrates, thereby contributing to the ability of ccRCC cells to resist glucose-deprivation conditions for 24 h [36]. Again, dapagliflozin has been demonstrated to be a potential therapeutic agent for RCC cell lines with wild-type VHL [16,30]. The study by Jang et al. was limited to the effects of high concentrations of dapagliflozin (20–100 μM) for 24 h [30]. In contrast, Kuang et al. evaluated the effects of lower concentrations (1, 2, and 4 μM) of dapagliflozin using an MTT assay for up to 72 h and an apoptosis assay at 2 μM for 48 h [16]. In the latter study, involving the use of ACNH and Caki-1 cells with wild-type VHL, an approximately 40% reduction in cell viability was observed with 2 μM dapagliflozin for 72 h in the MTT assay. Furthermore, the results of the apoptosis assay showed that dapagliflozin treatment increased the apoptosis rate by 1.89-fold compared to the control. Although a direct comparison of efficacy is not possible, these results may suggest a more rapid and potent effect compared to our findings using ccRCC cells with the VHL mutation. This discrepancy could be attributed to glucose-deprivation resistance associated with metabolic reprogramming in ccRCC cells with the VHL mutation. In contrast, the results of our study demonstrate that effective suppression can still be achieved in ccRCC cells with the VHL mutation through prolonged exposure to dapagliflozin for 96 h. Furthermore, our findings suggest that glucose uptake into *VHL* mutant ccRCC cells partially depends on SGLT2, which is vital in comprehending ccRCC biology. Although further investigations are necessary to assess the effects of other SGLT2is aside from dapagliflozin, our observations of the results of SGLT2 knockdown suggest SGLT2 targeting as a novel strategy against ccRCC.

The aging population and increasing prevalence of DM have led to a growing demand for safe and effective treatments in patients with ccRCC. SGLT2is are already sufficiently characterized in terms of their adverse event profiles, and no specific safety concerns have been reported regarding their use in combination with the standard-of-care regimens for patients with ccRCC, such as targeted therapies and immune checkpoint inhibitors, in real-world settings. SGLT2is, therefore, hold significant potential for drug repositioning as a therapeutic agent for ccRCC, and their use in combination with standard treatments may enhance treatment efficacy.

DM is associated with poor cancer-specific survival and overall survival in patients with ccRCC [11]. Because DM causes hyperglycemia and various other complications, the use of in vitro models eliminates potential confounders related to DM in vivo, enabling accurate assessment of the effects of glucose levels on ccRCC cell progression. Our use of media containing various concentrations of glucose demonstrates the importance of glycemic control in patients with ccRCC who suffer from DM. Notably, dapagliflozin inhibited ccRCC cell progression, even at low glucose concentrations, positioning SGLT2is as novel therapeutic agents in patients with and without DM.

## 4. Materials and Methods

### 4.1. Cell Culture and Chemicals

KMRC-1 and ACHN cell lines were obtained from the Japanese Collection of Research Bioresources Cell Bank (Osaka, Japan) and the American Type Culture Collection (Manassas, VA, USA), respectively. KMRC-1 is a human ccRCC line that contains the *VHL* mutation [37], and ACHN is a human RCC line with wild-type *VHL* [32]. Transduction-ready lentiviral particles containing three target-specific constructs encoding 19–25 nucleotides of shRNA (i.e., KMRC-1-SGLT2-shRNA and SGLT2 knockdown) or non-silencing control-shRNA (sc-108080; Santa Cruz Biotechnology) (i.e., KMRC-1-control-shRNA) were used to transfect KMRC-1 cells, as previously described [38,39]. All experiments were performed within 20 passages. KMRC-1, its sublines, and ACHN were maintained in Dulbecco’s modified Eagle’s medium (DMEM) with 25 mM glucose (Wako, Osaka, Japan) supplemented with 10% heat-inactivated FBS and penicillin (100 units/mL)–streptomycin (100 μg/mL). To perform further assays, the cells were cultured in 25 mM (450 mg/dL), 12.5 mM (225 mg/dL), or 5 mM (90 mg/dL) glucose medium containing 25 mM glucose or no glucose DMEM (Wako) supplemented with 10% heat-inactivated FBS and penicillin (100 units/mL)–streptomycin (100 μg/mL). The cells were maintained in an incubator at 37 °C under a humidified atmosphere of 5% CO_2_. Dapagliflozin was obtained from ChemScene (Monmouth Junction, NJ, USA).

### 4.2. Cell Viability

An MTT assay (Sigma-Aldrich, St. Louis, MO, USA) was used to evaluate cell viability. Cells (3–5 × 10^3^/well) were seeded in 96-well tissue culture plates and incubated for 96 h, followed by the addition of 10 μL MTT stock solution (5 mg/mL) to each well for 3 h at 37 °C. The medium was replaced with 100 μL dimethyl sulfoxide and incubated for 5 min at room temperature. Absorbance was measured at a wavelength of 570 nm, with background subtraction at 630 nm. All samples were tested in triplicate from the different wells.

### 4.3. Plate Colony Formation

A clonogenic assay was performed to evaluate clonogenic potential. Briefly, cells (1 × 10^3^/well) were seeded in six-well tissue culture plates and incubated until colonies were detected in the control well. Cells cultured in serum-free media or treated with 10 ng/mL recombinant human epidermal growth factor (R&D Systems, Minneapolis, MN, USA) served as negative and positive controls, respectively. After fixing the cells/colonies with methanol and staining with 0.1% crystal violet, each well was photographed to facilitate colony quantification using ImageJ software version 1.54 (National Institutes of Health, Bethesda, MD, USA). The assay was performed in triplicate.

### 4.4. Cell Migration

A scratch wound healing assay was adapted to evaluate cell migration. Cells were cultured to a density of ≥90% confluency in six-well tissue culture plates. Following manual scratching with a sterile 200 μL plastic pipette tip, the cells were incubated in serum-free DMEM for 24 h, fixed with methanol, and stained with 0.1% crystal violet. The wound width was examined and photographed using inverted microscopy, and the normalized cell-free area (24 h/0 h) was quantitated using ImageJ. The experiment was conducted in triplicate.

### 4.5. Apoptosis Analysis

A TdT-mediated dUTP nick end labeling (TUNEL) method was used to evaluate the degree of apoptosis (Apoptosis in situ Detection Kit; Wako, Osaka, Japan), followed by counterstaining for the nuclei with hematoxylin. Data were analyzed using ImageJ. The experiment was performed in triplicate.

### 4.6. Western Blot

Proteins were extracted with RIPA buffer containing a protease and phosphatase inhibitor cocktail (Halt Protease and Phosphatase Inhibitor Cocktail; Thermo Scientific, Rockford, IL, USA). A Simple Western system (ProteinSimple, Bio-Techne, Minneapolis, MN, USA) was utilized to detect protein signals [40]. The signals were visualized using a Wes system with Compass version 6.1.0 software (ProteinSimple). The primary antibodies used were as follows: anti-SGLT2 (clone D-6, 1:50; Santa Cruz Biotechnology, Dallas, TX, USA), anti-pVHL (clone Ig32, 1:25; BD Biosciences, Milpitas, CA, USA), and anti-GAPDH (clone G-9, 1:1000, Santa Cruz Biotechnology). The results were confirmed from at least three independent experiments.

### 4.7. Public Database Analysis

The R2 Genomics Analysis and Visualization Platform (https://hgserver1.amc.nl/cgi-bin/r2/main.cgi, accessed on 27 February 2025) was utilized to evaluate *SLC5A2* expression in ccRCC. The datasets were obtained from the Gene Expression Omnibus repository (accession numbers GSE53757 [18] and GSE11024 [19]).

### 4.8. Statistical Analysis

Student’s *t*-test was used for comparisons between two groups of nonparametric data. All in vitro assays were performed in at least three independent experiments. *p*-values < 0.05 were considered statistically significant.

## 5. Conclusions

The results of the present study demonstrate that SGLT2 inhibition suppresses the progression of ccRCC tumors, partly via apoptosis. This finding implies the potential application of SGLT2is for the treatment of ccRCC as a drug repositioning opportunity, and SGLT2is may also be a preferable treatment for patients with DM who have ccRCC. Further investigation into SGLT2is, including clarification of the mechanisms underlying their suppressive effects on tumor progression, is required to determine the biological significance of SGLT2 and its inhibitor in ccRCC.

## Data Availability

The data presented in this study are available from the corresponding author upon request but are not publicly available due to privacy and/or ethical restrictions.

## Abbreviations

The following abbreviations are used in this manuscript:

SGLT2	Sodium–glucose cotransporter 2
ccRCC	Clear cell renal cell carcinoma
VHL	von Hippel–Lindau
SGLT2i	Sodium–glucose cotransporter 2 inhibitor
RCC	Renal cell carcinoma
HIF	Hypoxia-inducible factor
DM	Diabetes mellitus
pVHL	von Hippel–Lindau protein
FBS	Fetal bovine serum
DMSO	Dimethyl sulfoxide
shRNA	Short hairpin RNA
DMEM	Dulbecco’s modified Eagle’s medium
